# Supplementary material for: Antibiofilm Activity of Amomum tsaoko Essential Oil on Staphylococcus aureus and Its Application in Pork Preservation
Source: Foods. 2025 Feb 15;14(4):662. doi: 10.3390/foods14040662 (PMC11853853; doi:10.3390/foods14040662)

Supplementary Material

Antibiofilm activity of *Amomum tsaoko* essential oil on *Staphylococcus aureus* and its application in pork preservation

Zhifeng Yan, Junrui Guo, Qiming Chen, Sibao Wan, Zhen Qin*, Haiyan Gao*

School of Life Sciences, Shanghai University, Shanghai, 200444, P. R. China

*Corresponding author.

E-mail address: gaohy@shu.edu.cn (H. Gao), qin_zhen@shu.edu.cn (Z. Qin)

Fax.: +86 021-66135172

No. 99 Shangda Road, Shanghai 200444, China

Table S1. Reverse transcription reaction system.

Compound	component content
Template RNA	500 ng
5× ATGScript® RT Mix	4 μL
RNase free ddH_2O	Guarantee system at 20 μL

Table S2. The cycle conditions used for real-time qRT-PCR.

Compound	component content
Hieff® qPCR SYBR Green Master Mix (No Rox)	10 μL
Primer	0.8 μL
Template cDNA	1 μL
Sterile water	Guarantee system at 20 μL

Figure S1. Effect of AEO on genes *atlA*, *sarA*, *and srtA* expression level. Control: a mixture of 1% (v/v) dimethyl sulfoxide and 0.1% (v/v) Tween 80. The experimental results are shown as the mean ± standard deviation (n = 3). Significant differences between groups are noted with different letters ($p < 0.05$).

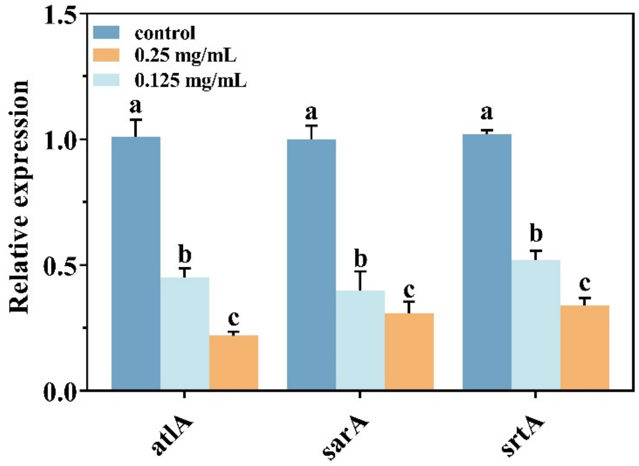

Table S3. Sensory evaluation of fresh pork.

Samples	Overall acceptability	Color	Flavor
Negative control	5.2 ± 0.41^{a}	4.9 ± 0.61^{a}	5.3 ± 0.62^{a}
2 mg/mL AEO	5.1 ± 0.49^{a}	5.2 ± 0.48^{ab}	5.3 ± 0.58^{a}
4 mg/mL AEO	5.4 ± 0.51^{ab}	5.4 ± 0.54^{ab}	5.4 ± 0.54^{a}
6 mg/mL AEO	5.6 ± 0.56^{ab}	5.6 ± 0.32^{ab}	5.6 ± 0.48^{a}
8 mg/mL AEO	5.9 ± 0.47^{ab}	6.2 ± 0.56^{b}	5.9 ± 0.35^{a}
10 mg/mL AEO	6.1 ± 0.41^{b}	6.3 ± 0.47^{b}	6.1 ± 0.44^{a}
Positive control	5.8 ± 0.56^{ab}	5.7 ± 0.48^{ab}	6.1 ± 0.49^{a}

All pork samples were stored at 4 °C for 10 d. Negative control: a mixture of 1% (v/v) dimethyl sulfoxide and 0.1% (v/v) Tween 80. Positive control: Kana solution (0.05 mg/mL). Significant differences between groups are noted with different letters ($p < 0.05$).

Figure S2. Weight loss (A) and Color analysis (B, C, D). All pork samples were stored at 4 °C for 10 d. Negative control: a mixture of 0.1% (v/v) Tween 80 and 1% (v/v) dimethyl sulfoxide. Positive control: Kana solution (0.05 mg/mL).

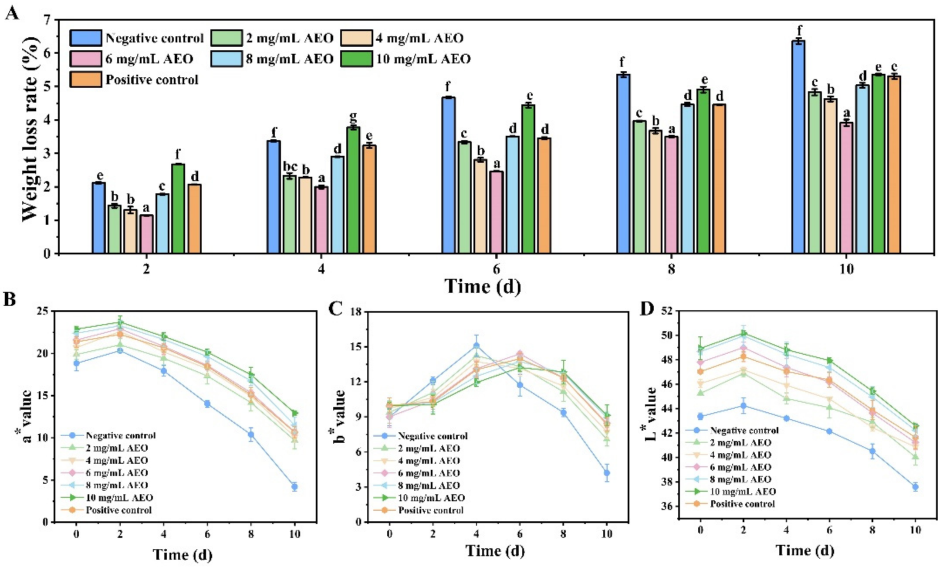

Figure S3. Photographs of different treatment groups stored for 0 d and 10 d. CK: a mixture of 0.1% (v/v) Tween 80 and 1% (v/v) dimethyl sulfoxide. Kana solution (10 mg/mL) and AEO (10 mg/mL).

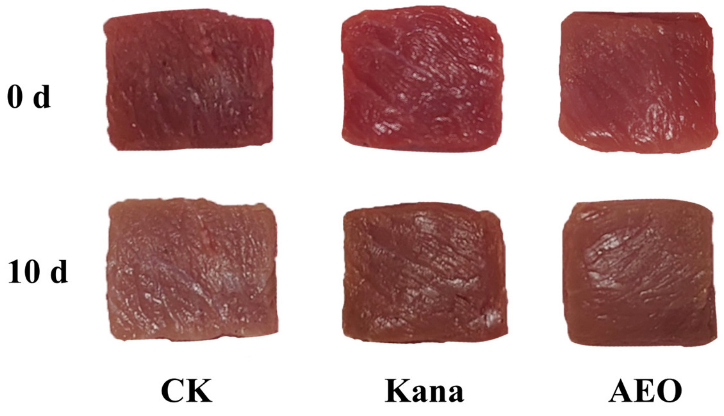

Supplement: Supplementary file 1 [file foods-14-00662-s001.zip › foods-3453579-supplementary.pdf]
